# The dual role of parasites and parasite-derived products in cancer biology: a systematic review of *in vitro* and *in vivo* evidence

**DOI:** 10.3389/fonc.2026.1894132

**Published:** 2026-07-16

**Authors:** Faisal Minshawi, Maimonah Alghanmi, Hassan Alwafi, Amal Mohammad Dustakir, Bayan Al Zoabi, Saleha Khan, Hattan S. Gattan, Isra M. Alsaady, Sarah A. Altwaim, Tope Oyelade, Ayat Zawawi

**Affiliations:** 1 Department of Clinical Laboratory Sciences, Faculty of Applied Medical Sciences, Umm Al-Qura University, Makkah, Saudi Arabia; 2 Department of Medical Laboratory Sciences, Faculty of Applied Medical Sciences, King Abdulaziz University, Jeddah, Saudi Arabia; 3 Vaccines and Immunotherapy Unit, King Fahd Medical Research Center, King Abdulaziz University, Jeddah, Saudi Arabia; 4 Faculty of Medicine, Umm Al-Qura University, Makkah, Saudi Arabia; 5 Clinical Laboratory Department, Makkah Medical Hospital, Makkah, Saudi Arabia; 6 General Medicine Practice Program, Batterjee Medical College, Jeddah, Saudi Arabia; 7 Special Infectious Agents Unit, King Fahd Medical Research Center, Jeddah, Saudi Arabia; 8 Department of Clinical Microbiology and Immunology, Faculty of Medicine, King Abdulaziz University, Jeddah, Saudi Arabia; 9 School of Medicine, Keele University, Keele, United Kingdom; 10 Division of Medicine, University College London, London, United Kingdom

**Keywords:** anticancer therapy, cancer biology, immune modulation, parasite-derived molecules, tumour microenvironment

## Abstract

Parasites and parasite-derived molecules play a complex role in cancer biology, and growing evidence suggests they influence cancer development. This systematic review explores the mechanisms underlying the pro-tumour and anti-tumour effects of parasites and their molecules in experimental cancer models. A total of 34 studies were reviewed, covering whole parasites and parasite-derived molecules, including vesicles, peptides, proteins, and miRNAs, across different cancer types. The evidence shows that the effects of parasites and parasite-derived molecules on cancer depend strongly on the parasite species, the molecules involved, and the host response. Pro-tumour effects were mainly associated with chronic inflammation, oxidative stress, epithelial proliferation, and immune modulation. In contrast, anti-tumour effects were associated with direct cytotoxicity, inhibition of migration, and immune activation. Several parasite-derived molecules exhibited anti-tumour effects beyond their usual sites of infection, suggesting broader biological activity and potential therapeutic relevance. In conclusion, this review highlights the dual, context-dependent influence of parasites and parasite-derived molecules on cancer progression and supports further mechanistic and translational studies to investigate their potential as cancer-promoting factors and anticancer therapeutic candidates.

**Systematic Review Registration:**

https://www.crd.york.ac.uk/PROSPERO/view/, identifier CRD420250656117.

## Introduction

Cancer continues to be the primary cause of mortality globally (1). Cancer arises through the accumulation of genetic and metabolic alterations that disrupt cellular pathways controlling proliferation, apoptosis, DNA repair, and immune surveillance (2). The cause of cancer involves a complex interplay between endogenous factors like mutations, hormonal influences, and metabolic disturbances, and exogenous factors such as environmental carcinogens, chronic inflammation, and infectious diseases (2, 3). Around 2.2 million new cancer cases globally are associated with viral and bacterial infections (4). Meanwhile, human parasitic infections are less appreciated (5). Infections can contribute to cancer development through various mechanisms, including chronic inflammation, pathogen-encoded oncoproteins that induce genomic alterations, and immunosuppression that diminishes tumour surveillance (6).

Parasites have been extensively studied to understand their relationship with their hosts. They remain prevalent in many endemic settings and are linked to cancer development (7). Parasites can promote cancer through chronic infections that trigger chronic inflammation, tissue damage, fibrosis, and immune dysregulation. One of their strategies for sustained presence with the host is to evade immune detection by secreting molecules that suppress the immune response (8). In this context, parasite-derived products could serve as potential candidates for treating autoimmune diseases and allergies (9). Our previous systematic review summarised evidence indicating that some helminth-derived proteins can reduce intestinal inflammation by upregulating anti-inflammatory cytokines, supporting T regulatory cell function, and improving outcomes in a colitis model (10). Parasites and their proteins can have anti-inflammatory effects, but this might be a double-edged sword in cancer. Immunomodulation may promote anti-tumour activity by regulating inflammation and shaping immune responses (11, 12), but it can also impair immune surveillance and reduce the immune system’s ability to recognise and eliminate malignant cells. However, limited studies have investigated whether these secretory molecules may directly interact with cancer cells, thereby increasing cell survival or promoting proliferation. Strong evidence indicates that cancer-promoting processes are present in *Schistosoma haematobium* (*S. haematobium*), *Opisthorchis viverrini* (*O. viverrini*), and *Clonorchis sinensis* (*C. sinensis*), all of which are associated with persistent infection and foster a microenvironment conducive to malignant transformation (13). On the other hand, some parasites or parasite-derived proteins, such as *Echinococcus granulosus* (*E. granulosus*), *Toxoplasma gondii* (*T.gondii*), and *Trypanosoma cruzi* (*T.cruzi*), can enhance the immune response against tumours, inhibit tumour growth, or mimic cancer antigens to stimulate the immune system (14).

The dual effect illustrates the complex interactions between parasites and cancer, involving chronic infection, host immune responses, tissue remodelling, and tumour microenvironment dynamics (13–16). Parasite-derived proteins have also been assessed *in vitro* for their effects on cancer cell cycle progression, apoptosis, motility, and cytokine responses under controlled conditions (17). This paradox underscores the biological and clinical significance of the association between parasites and cancer (4). Despite these observations of the paradoxical effect of parasites, they remain scattered across individual experimental models. Moreover, there remains a lack of comprehensive understanding of the common mechanistic pathways across diverse tumour types and parasitic species. Therefore, this study was conducted to evaluate the mechanistic pathways underlying both pro-tumour and anti-tumour effects of parasites or parasite-derived products across different experimental models and to identify shared versus tumour-specific biological patterns across cancer types.

## Methodology

### Search strategy and study selection

This systematic review was conducted and reported in accordance with the Preferred Reporting Items for Systematic Reviews and Meta-Analyses (PRISMA) 2020 statement (1) and adhered to the Meta-analysis of Observational Studies in Epidemiology (MOOSE) guidelines (2). The review protocol was prospectively registered with PROSPERO (Registration ID: 2025 CRD420250656117).

**Figure 1:**
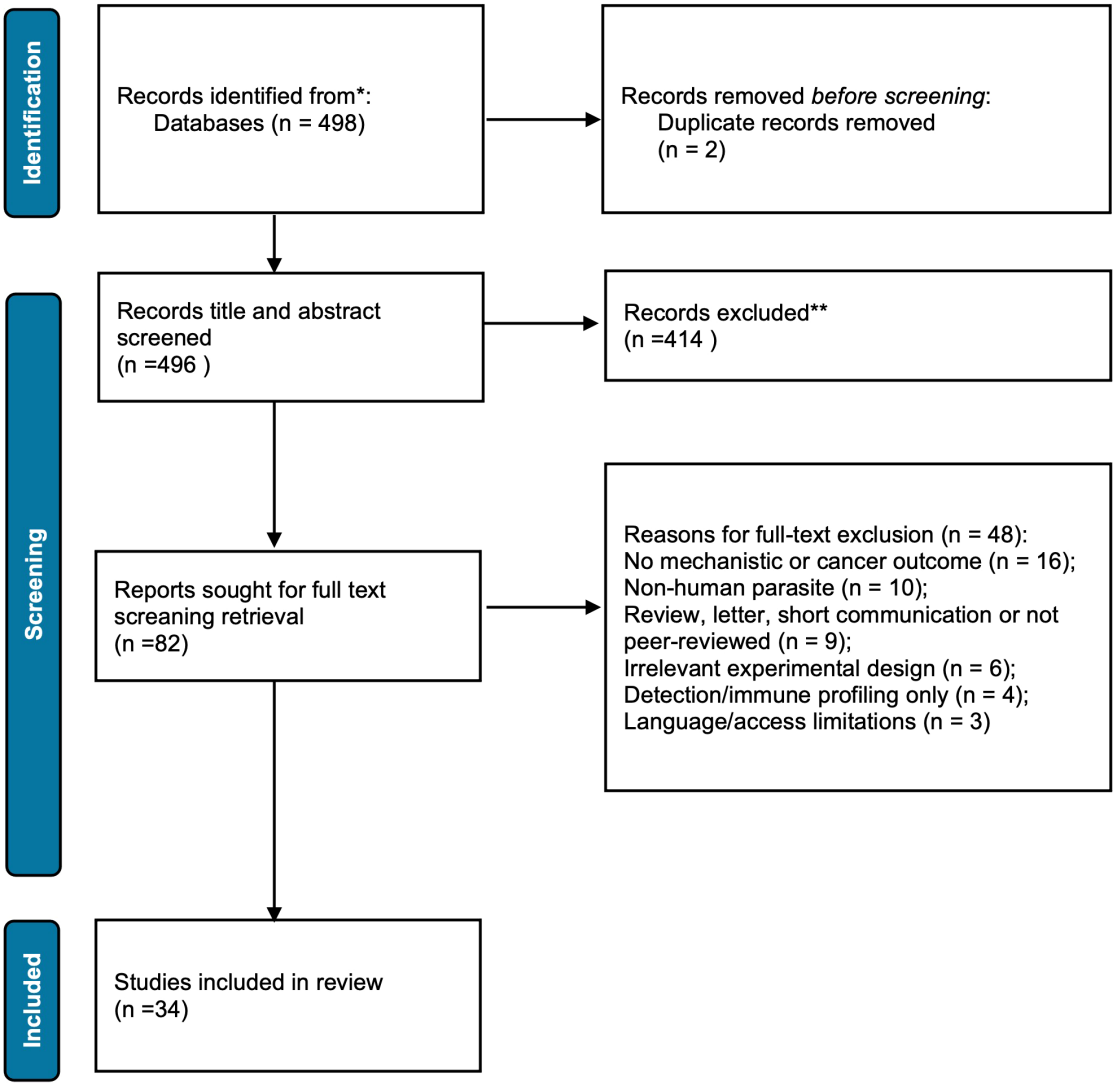
The PRISMA flow diagram of the study inclusion/exclusion process. * Medline, EMBASE, Web of Knowledge, Cochrane Library databases. ** Titled and abstract screening.

The literature search was conducted using PubMed/MEDLINE, EMBASE, and the Cochrane Library from the databases’ inception to January 2026 (**Figure 1**). Search terms combined controlled vocabulary (MeSH and Emtree) and free-text keywords related to parasites (e.g., parasite, helminth, protozoa), parasite-derived factors (e.g., excretory–secretory products, parasite-derived proteins, microRNA), and cancer-related outcomes (e.g., cancer, tumorigenesis, carcinogenesis, apoptosis, immune modulation, signalling pathways). Boolean operators, truncation, and alternative spellings were applied to maximise sensitivity.

### Eligibility criteria

Studies were eligible if they investigated the direct effects of parasites or parasite-derived molecules on cancer initiation, progression, or regression, using *in vitro* or *in vivo* models and reporting mechanistic or biological cancer-related outcomes (e.g., apoptosis, proliferation, tumour growth, or regression).

Exclusion criteria included review articles, editorials, conference abstracts, non–peer-reviewed publications, studies involving non-human parasites, irrelevant experimental models, absence of cancer-related outcomes, or studies limited to diagnostic or immune profiling without tumour endpoints. Articles unavailable in English or without an accessible full text were also excluded.

### Study selection

After removal of duplicate records (n = 2), 496 records were screened by title and abstract. Records not addressing parasite–cancer interactions or reporting non-original data were excluded. Eighty-two reports underwent full-text assessment for eligibility; 48 were excluded for predefined reasons (**Figure 1**). A total of 34 studies were included in the final review.

### Risk of bias evaluation

The risk of bias was independently evaluated utilising the SYRCLE Risk of Bias tool tailored for *in vivo* studies. The assessment encompassed key domains, including selection, performance, detection, attrition, reporting, and other biases. Each domain was classified as low, high, or unclear risk based on the methodological details provided. A total of twenty-three studies were examined. A high risk of bias indicated the potential presence of systematic errors, whereas a low risk suggested a robust methodological approach. An unclear risk signified insufficient information to determine bias. This evaluation underscores common limitations in experimental parasitology and cancer research, particularly in blinding, and highlights the need for improved reporting standards to enhance reproducibility and methodological validity.

### Data collection process and data synthesis

Data extraction was performed independently by five reviewers using a standardised extraction form. Discrepancies were resolved by consensus. Extracted data included publication details, parasite species or parasite-derived molecules, cancer type or experimental model, study design, biological source materials, proposed mechanisms of action, measured outcomes, molecular targets or pathways, and the reported effect on cancer progression (pro-tumorigenic or anti-tumorigenic).

## Results

### Characteristics of included studies

A total of 498 studies were identified, and 34 met the inclusion criteria for this systematic review (**Figure 1**). These studies evaluated the effects of parasites and parasite-derived products on cancer biology using *in vivo*, *in vitro*, and combined experimental approaches. This review showed a wide range of parasites and parasite-derived molecules, including *O. viverrini*, *S. haematobium*, *Schistosoma mansoni* (*S. mansoni*), *Schistosoma japonicum* (*S. japonicum*), *Trichinella spiralis* (*T.spiralis*), *T. gondii*, *T. cruzi*, *Cryptosporidium parvum* (*C.parvum*), *Blastocystis* spp.(*B.* spp.), *Blastocystis hominis* (*B. hominis*)*, C. sinensis*, *Toxocara canis* (*T. canis*), *P. falciparum* VAR2CSA, and *Anisakis* spp (*A.* spp.). The tested parasite-derived products include soluble antigens, excretory–secretory products, extracellular vesicles, lysate antigens, recombinant proteins, peptides, and parasite-derived non-coding RNAs.

The most studied cancer models include cholangiocarcinoma, colorectal, hepatocellular carcinoma, bladder cancer, melanoma, breast cancer, soft tissue sarcoma, and Burkitt lymphoma. The *in vivo* studies mainly used hamsters, mice, rats, SCID mice, nude mice, and tumour-bearing mouse models, whilst *in vitro* studies used various cancer cell lines, including CHO, H69, HCT116, HT-29, Caco-2, HepG2, Hepa1-6, SW620, and HUVECs.

In summary, the included studies provided a solid foundation for studying the mechanistic effects of parasite species and products on different cancer types using various experimental models.

### Quality of included studies

**Figure 2:**
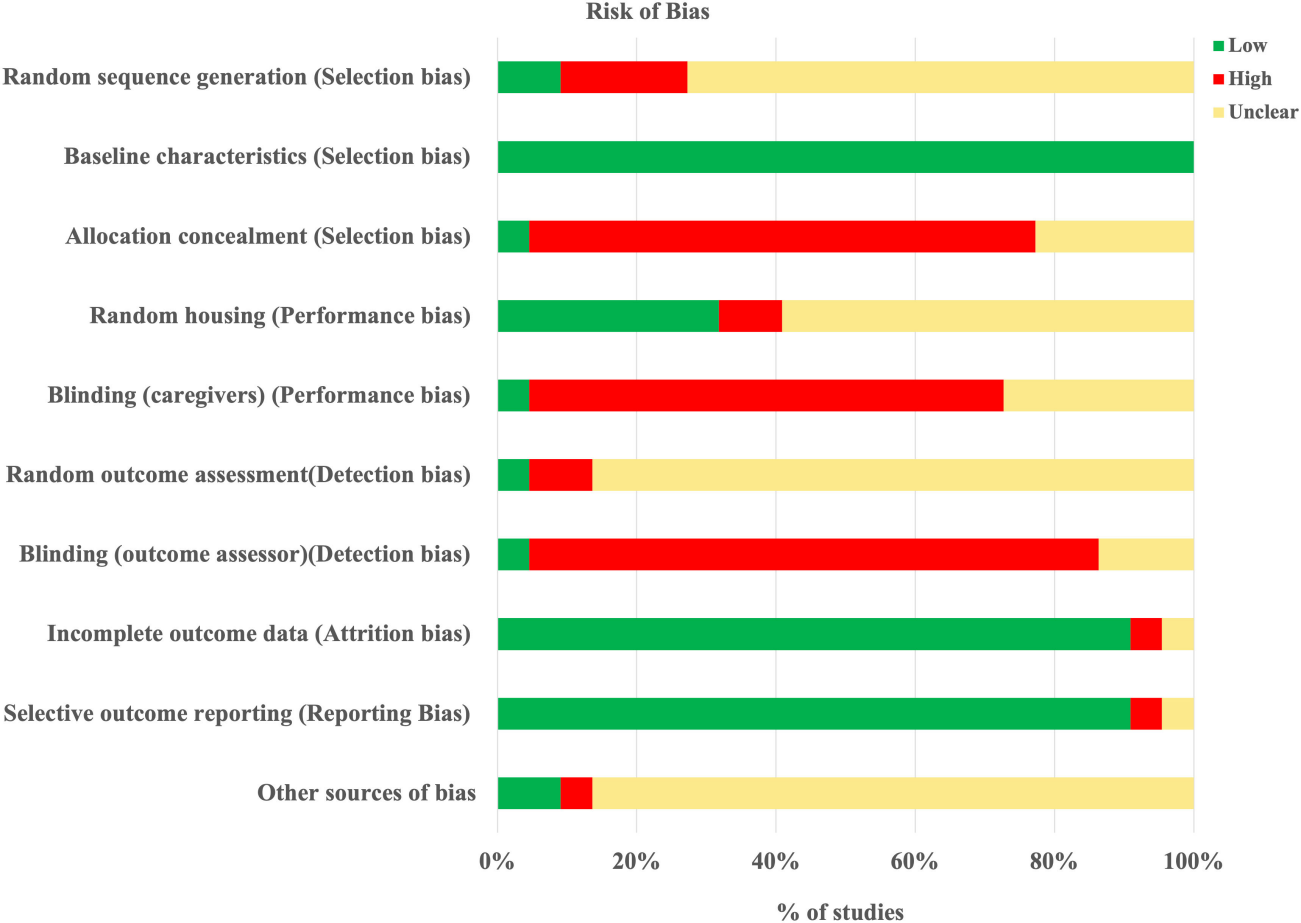
Risk-of-bias summary of the included studies according to SYRCLE domains. Stacked bars indicate the percentage of studies rated as low risk (green), high risk (red), or unclear risk (yellow) for each methodological domain.

The quality assessment showed variable risk of bias in animal studies (**Figure 2**). Most studies showed a low risk of bias for baseline characteristics, including incomplete outcome data and selective outcome reporting, indicating acceptable comparability between groups and limited concerns about missing data or selective reporting. On the other hand, several domains showed a high risk of bias due to methodological limitations, including potential selection, performance, and detection biases. In addition, insufficient reporting of key methodologies, such as random sequence generation, random housing, and random outcome assessment.

### The effects of parasites and parasite-derived proteins on tumour promotion and suppression

#### Overview of included studies

This systematic review included 34 studies. However, because some studies included both *in vivo* and *in vitro* experimental designs, these were analysed independently, yielding 38 experimental datasets for effect classification (**Table 1**). Overall, 26 of 38 datasets (68.4%) reported pro-tumour effects, whereas 12 of 38 datasets (31.6%) showed anti-tumour effects. In the *in vivo* studies, 17 of 23 studies (73.9%) showed pro-tumour activity, whilst 6 studies (26.1%) reported anti-tumour activity. Similarly, in the *in vitro* studies, 9 of 15 studies (60%) showed pro-tumour effects, whereas 6 studies (40%) showed anti-tumour effects.

**Table 1:** Characteristics of the included studies investigating parasite- and parasite-derived factors in cancer models.

Study	Year	Parasite/parasite product	Cancer type	Study type	Model/cell liens	Control
(18)	1978	*O. viverrini*	CCA	*In vivo*	Hamster	Untreated control; DMN alone control; Parasite alone control
(19)	1987	*O. viverrini*	CCA	*In vivo*	Hamster	Untreated control; Parasite-only controls (various doses); Carcinogen-only controls (various doses)
(20)	1988	*O. viverrini*	CCA and hepatocellular nodules	*In vivo*	Hamster	Untreated control; Nitrite control; Aminopyrine control; Parasite control
(21)	1988	*O. viverrini*	Liver and Pancreatic cancer	*In vivo*	Hamster	Basal diet control; BHA alone control; DHEA alone control; Parasite + diet controls
(22)	1992	*T. gondii-*lysate antigen	Soft tissue sarcoma	*In vivo*	Rat	Untreated tumour control; Saline control
(23)	1994	*O. viverrini*	CCA	*In vivo*	Hamster	DMN alone control; OV alone control; Untreated control
(24)	1996	*O. viverrini*	CCA and HCC	*In vivo*	Hamster	Untreated control (0 metacercariae); Noninfected control (BrdU analysis)
(25)	1998	*O. viverrini*	CCA and hepatocellular lesions	*In vivo*	Hamster liver tissue	Disease model control; Vehicle control
(26)	2001	*T. cruzi*	CRC	*In vivo*	Rat	Chemical carcinogen-treated control; non- immunised control
(27)	2009	*O. viverrini*	CCA	*In vivo*	Hamster/tissue	Normal control; NDMA alone control; Infection alone control
(28)	2009	*S. haematobium* antigen	Subcutaneous sarcoma	*In vivo*	nude mice	Untreated control animal group
(29)	2009	*S. haematobium* antigen	SCC	*In vitro*	CHO cells	Untreated control cells (multiple assays)
(30)	2011	*O. viverrini*	CCA	*In vivo*	Parasite infection model	Normal group; Infection alone group; NDMA alone group
(31)	2011	*S. haematobium* antigen	bladder cancer	*In vivo*	Mouse urothelium	Vehicle control (saline)
(32)	2012	*B. hominis* antigens	CRC	*In vitro*	HCT116 cells	Untreated control; Positive control (mitogen)
(33)	2012	*C. parvum*	Digestive adenocarcinoma	*In vivo*	SCID mouse	Negative control (PBS); Negative control (inactivated oocysts)
(34)	2013	*S. mansoni*	HCC	*In vivo*	Mouse	Control animals; Untreated control cells
(35)	2013	*S. haematobium-*SEA	Tumour-like phenotypes in urothelial cells	*In vitro*	HCV29 cells	S. mansoni infection only control; Healthy control
(36)	2015	*T. spiralis* and *T. spiralis-*ES L1 antigens	Melanoma	*In vitro/In vivo*	B16 cells/mouse	Uninfected control animals (in vivo); Untreated melanoma cells (in vitro)
(37)	2015	*O. viverrine-*Ov-GRN-1	CCA	*In vitro/In vivo*	Cell lines/mouse	Control recombinant protein (rTRX); Control dsRNA-treated flukes; Vehicle control (PBS); Baseline control
(38)	2015	*O. viverrine* EVs	CCA	*In vitro/In vivo*	H69 cells/mouse	Uninfected control hamsters; ES products control; BSA negative control; Medium alone control
(39)	2015	*B.spp.* ST-1	CRC	*In vitro*	HT-29 cells	Untreated control
(40)	2015	*P. falciparum*-VAR2CSA protein	BL	*In vitro*	BL cell lines/tissue	Endemic controls; Technical controls; Specificity controls; Protein control; Negative controls
(41)	2016	*O. viverrini* ES	CCA	*In vitro*	H69/CaCo-2 cells	Untreated control
(42)	2017	*O. viverrini* Ov-GRN-1	CCA	*In vitro*	HUVECs	Medium-alone control; Blank control; Negative control (anti-angiogenic); Positive control (pro-angiogenic)
(43)	2017	*C. sinensis* ES	CCA	*In vitro*	H69 cells	Untreated control; Vehicle control (PBS); Negative control siRNA; Liposome-only control
(44)	2018	*S. mansoni*-antigen extract and *T. spiralis*-antigen extract	CRC	*In vivo*	Mouse	Untreated control; Cancer control; ASMA control; ATSA control
(45)	2019	*S. japonicum* MicroRNA Sja-miR-3096 derived from	HCC	*In vitro/In vivo*	Hepa1-6/SMMC-7721/nude mice	Negative control mimics; Blank control; EV extraction control; Uninfected group; Empty plasmid control; Vehicle control (PBS); NC siRNA
(46)	2020	*T. canis*	Breast cancer	*In vivo*	4T1 mouse	Tumour-only control
(47)	2020	*S. mansoni* eggs + SEA (SEA) + IPSE/α-1	CRC	*In vitro*	SW620/hamster/biopsy	DMN alone control; OV alone control; Untreated control
(48)	2021	*S. japonicum* microRNA (sja-miR-61)	HCC	*In vitro/In vivo*	Hepa1-6/HepG2/HUVEC/mice	Negative control mimic; Mock control; NC siRNA; Empty vector control; NC-transfected cells; Uninfected mice
(49)	2021	*C. parvum*	CRC	*In vivo*	SCID mouse mice ileo-cecal tissue	Uninfected control
(50)	2024	*T. gondii-*GRA1-derived peptide	CRC	*In vitro*	Caco-2/HT29/HepG2	Carcinogen alone control; Parasite alone control; Untreated control
(51)	2024	*A. spp*-EVs	CRC	*In vitro*	intestinal organoids	PBS

TLA, T. gondii lysate antigen; DHEA, dehydroepiandrosterone; BHA, butylated hydroxyanisole; CHO, Chinese hamster ovary; SCID, severe combined immunodeficiency; Sh-SEA/SEA, soluble egg antigen; ESP, excretory-secretor; ES L1, excretory-secretory antigens from muscle larvae; EVs, extracellular vesicles; BL, Burkitt lymphoma; CCA, cholangiocarcinoma; CRC, Colorectal cancer; HCC, Hepatocellular carcinoma; SCC, Squamous cell carcinoma; HUVECs, human umbilical vein endothelial cells; NDMA, N-nitrosodimethylamine.

Mechanistically, the pro-tumour effects were grouped into four categories: coinfection or co-carcinogenesis, chronic infection-associated carcinogenesis, immune modulation that favours tumour progression, and activation of oncogenic signalling pathways. In contrast, the anti-tumour effects were classified into three main mechanisms: induction of apoptosis, inhibition of tumour migration and angiogenesis, and activation of anti-tumour immune responses.

#### *In vitro* effects of parasites and parasite-derived proteins on tumour promotion and suppression

The analysis of 15 *in vitro* studies (**Table 2**) revealed a complex, often dichotomous relationship between parasitic organisms and cancer, demonstrating that parasite-derived molecules can promote or suppress tumorigenic phenotypes. The majority of the studies (9 out of 15) showed a pro-tumorigenic effect through immune modulation and activation of oncogenic pathways.

**Table 2:** *In vitro* studies of parasite and parasite-derived proteins on cancer.

Pro-tumour effects (n = 9 studies)						
Ref	Parasite/protein	Target cancer (Clinical)	Cell line/model	Key mechanisms	Outcomes measured	Molecular targets
Immune modulation						
(32)	B. hominis antigens	Colorectal cancer	HCT116 cells	↑Proliferation, ↑ Th2 cytokines expression	MTT viability, gene expression	↑TNF-kB, ↑Th2 cytokines, ↑Cathepsin B
Activation of oncogenic pathways						
(37)	O. viverrini Ov-GRN-1	Cholangiocarcinoma	H69 cholangiocytes	↑Proliferation,	Scratch assays, proliferation, migration	↑CXCL1/2/8-CXCR2- EGFR-MAPK axis
(38)	O. viverrini EVs	Cholangiocarcinoma	H69 cholangiocytes	EV internalization, ↑proliferation	Cell proliferation, IL-6 secretion	↑MAPK signalling, ↑IL-6
(41)	O. viverrini ES products	Cholangiocarcinoma	H69, Caco-2 cells	↑Glycolysis, ER stress	Proteomic analysis	Glycolysis/gluco- neogenesis pathways
(42)	O. viverrini Ov-GRN-1	Cholangiocarcinoma	HUVECs	↑Angiogenesis, ↑endothelial proliferation	Proliferation, tubule formation	Angiogenesis stimulation
(43)	Clonorchis sinensis ESP + NDMA	Cholangiocarcinoma	H69 cholangiocytes	↑Proliferation, G2/M phase shift	Cell proliferation, cell cycle	↑Connexin 43/26, ↑E2F1, ↑Ki-67
(47)	S. mansoni eggs/SEA/IPSE	Colorectal cancer	SW620 cells	Wnt/β-catenin activation, JNK/c-Jun pathway	β-catenin, AP-1 reporter, proliferation	↑Wnt/β-catenin, ↑ c-Jun/AP-1, ↑Cyclin D1
(29)	S. haematobium total antigen	Squamous cell carcinoma	CHO cells	↑Proliferation, ↑migration, ↑invasion, ↓apoptosis	Cell proliferation, apoptosis, migration, invasion	↑Bcl-2, ↓p27, cell cycle disruption
(51)	Anisakis spp. EVs	Colorectal cancer	Human intestinal organoids	↓Tumour suppressors	RNA-seq, qRT-PCR, cytokine profiling	↓EPHB2, ↓LEFTY1, ↑NUPR1

Anti-tumour effects (n = 6 studies)						
Ref	Parasite/protein	Target cancer (clinical)	Cell line/model	Key mechanisms	Outcomes measured	Molecular targets
Direct tumour cell cytotoxicity and growth suppression						
(36)	T. spiralis ES L1	Melanoma	B16 melanoma cells	↑Apoptosis (extrinsic pathway)	Cell survival, apoptosis assays	↑Caspase-3, ↑Caspase-8
(39)	B.spp. ST-1	Colorectal cancer	HT-29 cells	Retinoic acid pathway modulation	Gene expression arrays, qRT-PCR	↑CRABP2, ↓PCNA, ↑RARα
(40)	P. falciparum VAR2CSA	Burkitt lymphoma	Burkitt lymphoma cell lines	Oncofetal chondroitin sulphate targeting	Binding assays, cytotoxicity	Oncofetal chondroitin sulphate A
(50)	T. gondii peptides	Colorectal + Hepatocellular carcinoma	Caco-2, HT29, HepG2	↑Apoptosis	MTT viability, flow cytometry	↓Bcl-2, ↓APAF1, cytochrome c/caspase cascade
Inhibition of migration						
(45)	S. japonicum miR-3096	Hepatocellular carcinoma	Hepa1-6, SMMC-7721	↓Proliferation, ↓migration, G0/G1 arrest	Proliferation, colony formation, migration	↓PIK3C2A -> ↓mTOR signalling
(48)	S. japonicum miR-61	Hepatocellular carcinoma	Hepa1-6, HepG2, HUVECs	↓Migration, ↓angiogenesis	Migration assays, angiogenesis markers	↓PGAM1 (glycolytic enzyme)

*B. hominis* antigens enhanced the proliferation of colorectal cancer cells and the expression of Th2-associated cytokines, indicating a pro-tumour immunomodulatory effect (32). Four studies examined the pro-tumorigenic effects of *O. viverrini*-derived products on biliary and endothelial cell lines. *O. viverrini* extracellular vesicles were internalised by H69 cholangiocytes, driving cell proliferation and Interleukin-6 (IL-6) secretion through MAPK signalling activation (38). Another study reported that *O. viverrini* Ov-GRN-1 promoted both proliferation and migration of H69 cholangiocytes through the CXCL1/2/8-CXCR2-EGFR-MAPK signalling axis, as confirmed by scratch and proliferation assays (37). A subsequent proteomic study demonstrated that *O. viverrini* excretory-secretory products reprogrammed metabolism in H69 and Caco-2 cells, inducing endoplasmic reticulum stress and upregulating glycolysis and gluconeogenesis pathways (41). *O. viverrini* Ov-GRN-1, extracellular vesicles, and ES products promoted cholangiocyte proliferation, migration, angiogenesis, and metabolic reprogramming (42). Moreover, *C. sinensis* excretory-secretory products and the carcinogen NDMA induced cell-cycle progression by upregulation of Ki-67, E2F1, and connexin 43/26 (43). *S. mansoni* eggs, soluble egg antigen, and IPSE activated the Wnt/β-catenin and JNK/c-Jun pathways in SW620 colorectal cancer cells through activated Wnt/β-catenin and JNK/c-Jun signalling (47). *S. haematobium* total antigen enhanced proliferation and migration whilst reducing apoptosis in CHO cells, whilst suppressing apoptosis, mediated through Bcl-2 upregulation, p27 downregulation, and cell cycle disruption (29). Using human intestinal organoids, *Anisakis* extracellular vesicles suppressed tumour suppressor pathways by downregulating the tumour suppressors EPHB2 and LEFTY1 and upregulating NUPR1 (51).

Conversely, a subset of studies highlights the potent anti-tumorigenic activities of parasite-derived molecules (6 out of 15). ES products from the nematode *T. spiralis*, for example, induce apoptosis in melanoma cells via the extrinsic pathway, involving the activation of caspases 8 and 3 (36). In a similar vein, peptides derived from *T. gondii* trigger mitochondrial-dependent apoptosis in colorectal and liver cancer cell lines by downregulating Bcl-2 and upregulating the apoptosis-promoting factor APAF1 (50). Research on *S. japonicum* has identified specific microRNAs with significant anti-cancer properties (45, 48). Notably, Sja-miR-3096 inhibits hepatoma cell proliferation and induces G0/G1 cell cycle arrest by targeting PIK3C2A, thereby suppressing the mTOR signalling pathway. Another microRNA, sja-miR-61, inhibits hepatoma cell migration and angiogenesis by directly targeting phosphoglycerate mutase 1 (PGAM1). In colorectal cancer models, lysates from *B.spp.* ST-1 were found to modulate retinoic acid signalling pathways by upregulating CRABP2 and RARα, which, in turn, led to downregulation of the proliferation marker PCNA and reduced cell growth (39). The VAR2CSA, a surface protein of *P. falciparum*, selectively bound Burkitt lymphoma cell lines via oncofetal chondroitin sulphate, thereby demonstrating direct tumour cell killing by exploiting the aberrant re-expression of this placenta-restricted glycosaminoglycan on malignant lymphoma cells (40).

In summary, *in vitro* evidence underscores the context-dependent and molecule-specific roles of parasitic factors in carcinogenesis. While many parasite-derived components clearly promote established hallmarks of cancer, including sustained proliferation, angiogenesis, and immune evasion, others exhibit strong antiproliferative and proapoptotic effects. This dual functionality suggests that specific parasite molecules could serve not only as etiological agents in infection-associated cancers but also as a novel source of targeted therapeutic agents.

#### *In vivo* effects of parasitic infection and parasite-derived molecules on cancer

The *in vivo* studies comprise 23 studies utilising different murine and hamster models (**Table 3**). Most *in vivo* studies reported tumour-promoting outcomes (17 of 23), frequently in the presence of chemical or viral cofactors. *O. viverrini* infection was consistently associated with cholangiocarcinoma in animal models, with tumour incidence approaching 100% in Syrian golden hamsters co-exposed to carcinogens such as dimethylnitrosamine (DMN) or N-nitrosodimethylamine (NDMA) (18–20, 23–25). Pathological features included chronic inflammation, cholangiofibrosis, and epithelial dysplasia, accompanied by activation of Wnt/β-catenin and MAPK signalling, and increased expression of ERBB2 and Transforming Growth Factor-Beta1 (TGF-β1). Chronic infection with *S. haematobium* (31) and *S. mansoni* (35) induces urothelial inflammation and dysplasia in bladder tissues and hepatocellular carcinoma, respectively, in a murine model. This effect is mainly observed through the generation of reactive oxygen and nitrogen species (ROS/RNS)- mediated DNA damage, including P53 gene mutations.

**Table 3:** *In vivo* studies of parasite and parasite-derived proteins on cancer.

Pro-tumour effects (n = 17 studies)						
Ref	Parasite/protein	Cancer type/model	Animal model	Key mechanisms	Outcomes measured	Molecular Targets
Chronic Infection and co-carcinogenesis						
(18)	*O. viverrini*	Cholangiocarcinoma	Syrian golden hamsters	Synergistic carcinogenesis with DMN	Cholangiocarcinoma incidence (100%)	DMN acts on parasite-altered epithelium
(19)	*O. viverrini*	Cholangiocarcinoma	Syrian golden hamsters	Dose-dependent tumour promotion	Cholangiocarcinoma incidence, histopathology	Parasite-enhanced DMN carcinogenesis
(20)	*O. viverrini*	Cholangiocarcinoma and HCC	Syrian golden hamsters	Synergistic effect with nitrite/aminopyrine	Tumour incidence, histopathology	Nitrosamine formation with inflammation
(23)	*O. viverrini*	Cholangiocarcinoma	Syrian golden hamsters	Chronic inflammation, epithelial proliferation	Cholangiocarcinoma incidence/severity	DMN and parasite-induced inflammation
(24)	*O. viverrini*	Cholangiocarcinoma and HCC	Syrian golden hamsters	Chronic irritation, increased cell turnover	Proliferative lesions, cholangiofibrosis	Tumour-promoting chronic inflammation
(25)	*O. viverrini*	Cholangiocarcinoma and HCC	Syrian golden hamsters	Parasite clearance effects, chemoprevention	Lesion counts, liver weights	Inflammatory modulation, DHEA pathway
(31)	*S. haematobium*	Bladder cancer	CD-1 mice	Chronic inflammation, ROS/RNS generation	Urothelial dysplasia, inflammatory infiltrate	Reactive oxygen/nitrogen species
(35)	*S. mansoni*	Hepatocellular carcinoma	Swiss albino mice	Synergistic hepatocarcinogenesis with DEN	Liver dysplasia, AFP/ferritin, survival	Chronic inflammation, p53 mutation
(33)	*C. parvum*	Digestive adenocarcinoma	CB17-SCID mice	Chronic infection, epithelial transformation	Neoplastic lesions, parasite burden	Infection-driven proliferation, immune evasion
Immune modulation						
(21)	*O. viverrini*	Liver and Pancreatic cancer	Syrian golden hamsters	Immune modulation, GST-P induction	Lesion count, GST-P expression	GST-P expression, bile duct proliferation
(28)	*S. haematobium* total antigen	Subcutaneous sarcoma	Nude mice xenograft (subcutaneous CHO inoculation)	Schistosomal antigens → fibroblast stimulation → sarcoma formation	Gross tumourigenesis	Granuloma → cytokines/growth factors → fibroplasia
(46)	*T. canis*	Breast cancer	BALB/c mice	Tumour immune microenvironment modulation	Tumour size/weight, immune populations	↑Th2, macrophages, ↓CD8+ T cells
(49)	*C. parvum*	Colorectal cancer	CB17-SCID mice	Immune modulation, tumour microenvironment	Adenocarcinoma development, gene expression	↓α-defensins, Wnt/NF-κB/Hedgehog pathways
Activation of oncogenic pathways						
(27)	*O. viverrini*	Cholangiocarcinoma	Syrian golden hamsters	RB pathway disruption	Gene expression, histopathology	↓RB1, ↓p16INK4, ↑cyclin D1, CDK4
(30)	*O. viverrini*	Cholangiocarcinoma	Syrian golden hamsters	Dysregulated gene expression, oncogenesis	Transcriptomic profiling, histology	↑ERBB2, ↑MMP9, ↑TGF-β1, MAPK/Wnt signalling
(37)	*O. viverrini* Ov-GRN-1	Cholangiocarcinoma	BALB/c mice	Epithelial proliferation, angiogenesis	Wound closure, angiogenesis, gene expression	↑CXCL1/2/8–CXCR2–EGFR–MAPK axis
(34)	*S. haematobium*	Bladder cancer	CD-1 mice	Oestrogen-DNA adducts, oxidative stress	Proliferation, apoptosis, DNA damage	Catechol-oestrogens, DNA adduct formation

Anti-tumour effects (n = 6 studies)						
Ref	Parasite/protein	Cancer type/model	Animal model	Key mechanisms	Outcomes measured	Molecular targets
Apoptosis						
(36)	*T. spiralis* ES L1	Melanoma	C57BL/6 mice	Apoptosis induction, necrosis suppression	Tumour volume, necrosis/apoptosis %	Caspase-3/8 dependent apoptosis
Inhibition of migration						
(45)	*S. japonicum* miR-3096	Hepatocellular carcinoma	BALB/c nude mice	miR-3096 suppresses via PIK3C2A/mTOR	Tumour volume/weight reduction	↓PIK3C2A → ↓mTOR signalling
(48)	*S. japonicum* miR-61	Hepatocellular carcinoma	C57BL/6J mice	Migration/angiogenesis inhibition via PGAM1	Tumour volume/weight, CD34, migration	↓PGAM1, anti-angiogenesis
Immune activation						
(22)	*T. gondii* TLA	Fibrosarcoma	Wistar rats	Immune modulation, Thy-1+ cell activation	Tumour growth inhibition, immune infiltration	Thy-1+ cells, host immune response
(26)	*T. cruzi*	Colorectal cancer	Wistar rats	Immune modulation, host resistance	Colon tumour incidence, histopathology	Systemic immune modulation
(44)	*S. mansoni* and *T. spiralis*	Colorectal cancer	Swiss albino mice	Immune modulation, regulatory T cells	Tumour size, cytokine levels, Treg cells	↑FoxP3+ Tregs, ↓IL-17, ↑IL-10

In addition, 4 studies showed that immunomodulation promotes carcinogenesis, including *O. viverrini* (35), *S. haematobium* total antigen (28), *T. canis* (46), and *C. parvum* (49). *O. viverrini* promoted liver and pancreatic lesions with increased GST-P expression, which is an immune modulatory effect, and bile duct proliferation, whilst *S. haematobium* tatal antigen enhanced sarcoma formation through fibroblast stimulation, granuloma-related cytokines, and fibroplasia. *T. canis* induces breast cancer by shifting the tumour microenvironment towards Th2 responses and suppressing CD8+ T cells. In addition, *C. parvum* promoted CRC development in SCID mice, leading to alterations in immune and cancer-related signalling pathways.

Moreover, some parasites may promote tumour progression by activating or disrupting cancer-related signalling pathways. *O. viverrini* was linked to cholangiocarcinoma through RB pathway disruption, reduced RB1 and p16INK4 expression, and increased cyclin D1/CDK4 activity (27). Other *O. viverrini* models showed dysregulated oncogenic gene expression involving ERBB2, MMP9, TGF-β1, MAPK, and Wnt signalling (30). *S. haematobium* promoted bladder cancer through oestrogen-DNA adduct formation, oxidative stress, and DNA damage (34). In addition, *O. viverrini* Ov-GRN-1 enhanced epithelial proliferation and angiogenesis through the CXCL1/2/8–CXCR2–EGFR–MAPK axis. Overall, these results indicate that parasite-associated oncogenic signalling may drive proliferation, DNA damage, angiogenesis, and malignant transformation (37).

A limited number of *in vivo* studies (6/23) showed antitumour activity through apoptosis, migration inhibition, and immune activation. *T. spiralis* ES L1 reduced melanoma growth by inducing caspase-3/8–dependent apoptosis (36). *S. japonicum* miR-3096 and miR-61 inhibited hepatocellular carcinoma growth by reducing tumour volume, migration, and angiogenesis through suppression of PIK3C2A/mTOR and PGAM1-related pathways (45, 48). In addition, *T. gondii* TLA (22), *T. cruzi* (26), and combined *S. mansoni*/*T. spiralis* (44) exposures showed anti-tumour activity through an immunoregulatory mechanism in which FoxP3+ Treg expansion and Interleukin-10 (IL-10) elevation suppressed pro-tumorigenic chronic colonic inflammation, as evidenced by a significant reduction in IL-17.

In summary, most findings supported a pro-tumour effect of parasite infection. The *in vivo* evidence indicates that chronic infection, persistent inflammation, oxidative stress, and immune deregulation are the major drivers of parasite-induced tumour progression. On the other hand, limited studies found that parasite-derived products may exert antitumour effects by acting as immunomodulatory factors that suppress tumour growth, enhancing immune cell infiltration and inducing molecular mimicry or cross-reactivity against tumour-associated antigens.

## Discussion

**Figure 3:**
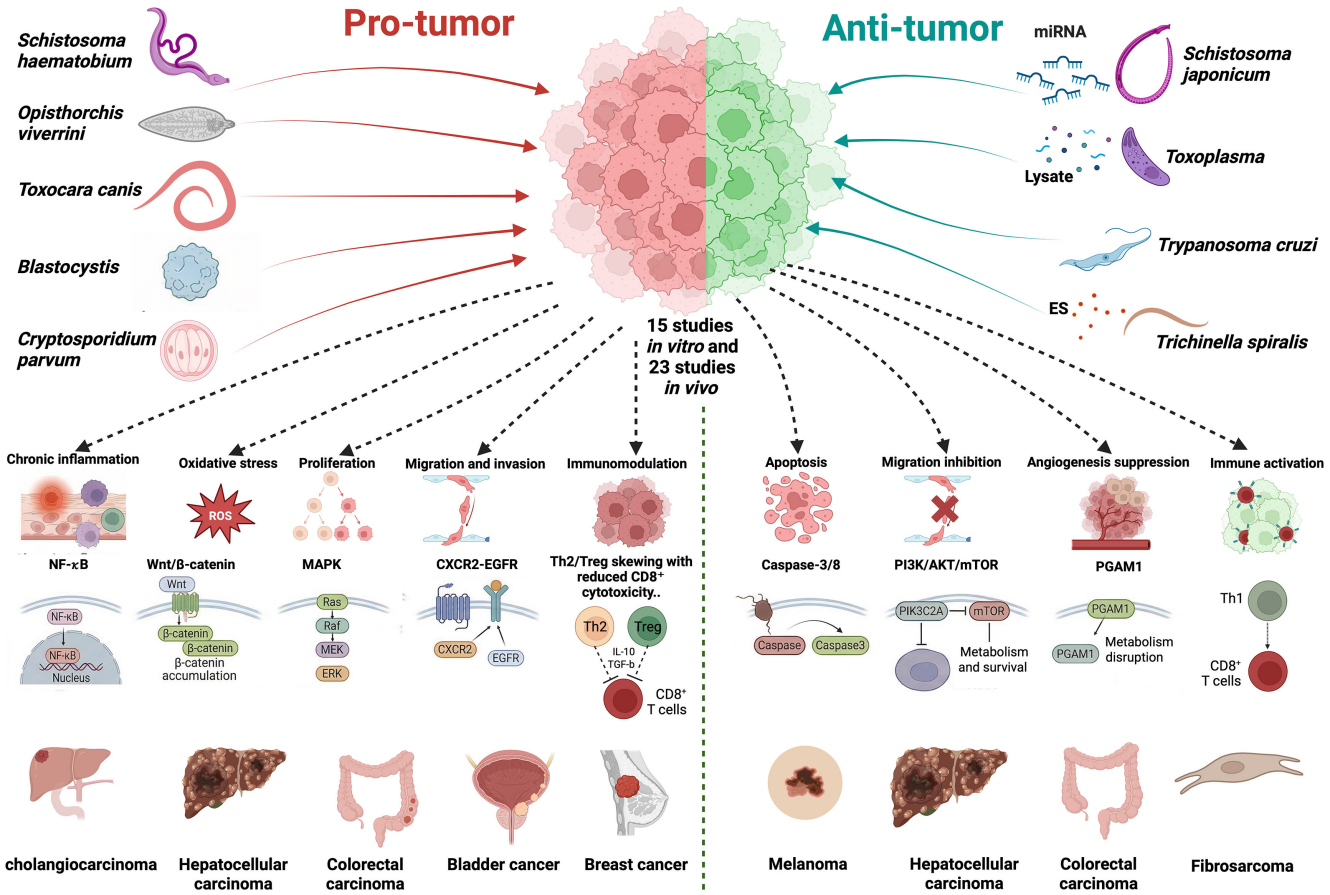
Dual role of parasites and parasite-derived proteins in cancer biology. The figure summarises the effects of parasites and parasite-derived molecules on tumour progression or regression. Created in BioRender. Minshawi, F. (2026) https://BioRender.com/eeysm78.

This study aimed to examine the mechanisms underlying the dual effects of parasites and their products on cancer across different experimental models. The results reveal a dual role in cancer biology. Observations from 34 studies suggest that both protumour and antitumour effects are influenced by parasite dependence, chronicity, and the host environment (**Figure 3**). The studies showed that the parasites may promote cancer progression through various mechanisms, including chronic inflammation, co-infection, co-carcinogenesis, and immunomodulation. Conversely, they exert anti-tumour effects through direct cytotoxicity, immune activation and surveillance, and molecular modulation.

This study highlights that chronic infection acts as a direct carcinogen. For instance, chronic infection of *S. haematobium* promotes bladder carcinoma, and *S. mansoni* promotes hepatocellular carcinoma through sustained ROS/RNS-mediated genotoxic damage, demonstrating that protracted parasite-driven inflammation is sufficient to initiate malignant transformation independently of other risk factors (31, 34, 35). Several *in vitro* studies provide molecular evidence of the specific signalling pathways, such as MAPK, NF-κB, Cyclin D1, and MCM2, that drive neoplastic transformation. This molecular profile may promote cell cycle progression, DNA replication, and resistance to apoptosis (52–54). Moreover, the co-infection context further amplifies the inflammatory cascade. A previous study reviewed the mechanism by which *O. viverrini* infection interacts with bacterial infection (*Helicobacter pylori*), thereby increasing the risk of developing CCA (55). *P. falciparum* promotes the expansion of Epstein-Barr virus (EBV)-infected B-cells, enhances AID-driven DNA damage, and raises the risk of c-MYC translocation, all of which contribute to the development of Burkitt’s lymphoma (56). In addition, this study suggests that parasitic infection could accelerate the development of cancer in the presence of carcinogens. Here we report that early studies on *O. viverrini* act synergistically with nitrosamine carcinogens, including DMN and NDMA, to induce tumours. NDMA has emerged as a major environmental contaminant, originating from both human activities and natural sources (57, 58). Ten studies published between 1978 and 2008 showed that co-exposure to *O. viverrini* and nitrosamine carcinogens consistently induced cholangiocarcinoma in animal models, with tumour incidence reaching 100%, whereas exposure to either agent alone was insufficient to produce comparable carcinogenic effects (18–21, 23–25, 27, 30, 59). This data indicates that parasites might not primarily act as carcinogenic initiators but rather as powerful tumour promoters that enhance the oncogenic potential of pre-existing DNA damage.

The oncological effects of parasitic infection vary and are not predictable. They depend on the parasite species, duration of infection, and the host’s immune response. Persistent Th2 environments tend to promote tumour growth, whilst acute Th1-polarised responses activate anti-tumour immunity (60). A previous review sharpens this picture by concluding that cancer cells mirror helminth survival strategies such as immunoevasion, metabolic adaptation, tissue remodelling, and host manipulation, which partly explains why those responses can be either tumoricidal or tolerogenic (61). This study summarised that the pro-tumour effects were associated with inflammatory and immunoregulatory changes, including increased Th2 responses, macrophage involvement, reduced CD8+ T-cell activity, and remodelling of the tumour microenvironment, as reported with *B. hominis*, *O. viverrini* (21)*, T. canis* (46), *Anisakis* spp (51), and *C. parvum* (33). Parasite-associated immune environments recruit and activate the same cellular components as those involved in cancer-associated immune remodelling: group 2 innate lymphoid cells (ILC2s), M2-polarised macrophages, eosinophils, and Tregs. During chronic parasitic infection, epithelial cells release IL-33, TSLP, and IL-25, which sustain ILC2 activation and Th2 differentiation, thereby generating a cytokine environment dominated by IL-4, IL-5, and IL-13 (62). This drives M2 macrophage polarisation, marked by arginase-1, CD163, and CD206, and is associated with downstream production of TGF-β, VEGF, and CCL17/CCL22. Within the tumour microenvironment, M2-polarised Tumour-Associated Macrophages (TAMs) facilitate vascular remodelling, extracellular matrix turnover, and suppression of CD8+ T cells through mechanisms nearly identical to the wound-healing programmes parasites exploit for persistence (63–65). ILC2-derived IL-13 amplifies M2 phenotype and expands Myeloid-derived suppressor cells (MDSCs); parasite-induced Tregs, acting through IL-10 and TGF-β, further suppress checkpoint responsiveness. Fibroblast activation, collagen deposition, and sustained angiogenesis are shared features of chronic parasitic infection and tumour stroma alike, which explains the resemblance that persistent type 2 environments are permissive for tumour growth (66). However, the prevailing assumption that Th2 immunity promotes tumorigenesis has been challenged. Wagner, Nishikawa, and Koyasu reviewed that Th2 responses can, depending on host and microenvironmental context, actively suppress tumour (60). For instance, IL-5-recruited eosinophils can kill tumour cells directly through major basic protein, eosinophil peroxidase, and granzyme A (67), and their infiltration correlates with favourable prognosis in colorectal cancer (68, 69). Here, we highlighted that antitumour effects were associated with immune activation, immune cell infiltration, and enhanced host resistance, particularly in studies involving *T. gondii* (22) and *T. cruzi* (26). Th2 cytokines also promote eosinophil recruitment to tumours (70), and IL-4 deficiency impairs tumour clearance in experimental models (71). ILC2s, as a primary source of tumour-associated IL-4, can support CD8+ cytotoxic function, as demonstrated by IL-4 signalling via STAT6 and mTOR revitalising exhausted CD8+ T cells in the tumour microenvironment (72). This functional plasticity is precisely what underlies the species- and host-dependent paradox described in this review.

In addition, the reported modulation of FoxP3+ regulatory T cells, IL-10, and IL-17 in *S. mansoni* and *T. spiralis* models suggests that parasite-driven immune regulation can shift the tumour environment towards either tumour promotion or tumour suppression, depending on the parasite species, cytokine profile, and experimental cancer model. (44). Parasites that induce a chronic environment dominated by IL-10 and TGF-β reinforce tumour immune evasion strategies. In contrast, parasites that are associated with acute or self-limiting infections can promote anti-tumorigenic immune responses (61, 73). In the context of IL-10, for example, our previous study concluded that IL-10 may have a dual role in cancer biology, possibly due to the heterogeneity of CRC, which encompasses subtypes defined by molecular signatures. (74). Interpreting parasite-cancer interactions therefore requires identifying not only which organism is involved but also which type 2 immune modules are active, in which tissue compartment, and at which stage of tumour development.

Parasite-derived molecules may exhibit anti-tumour activity that differs from that observed during natural infection, as isolated parasite products can selectively modulate immune responses without inducing the chronic inflammation or tissue damage associated with live infection. For example, *S. japonicum* infection can influence macrophage polarisation, shifting from M1 during acute infection to M2 in chronic stages (75). Additionally, schistosome worm antigen (SWA) promotes M1, whereas schistosome soluble egg antigen (SEA) favours M2, depending on the infection stage and the antigen involved (75). However, *S. japonicum* non-coding RNA, such as miR-3096 and miR-61, shows anti-tumour effects by inhibiting migration (45, 48). Another example is seen in *P. falciparum* malaria infection, which can activate B cells through PfEMP1/CIDR1α, promoting polyclonal B-cell expansion and increasing the pool of EBV-infected B cells at risk of malignant transformation (76). However, using *P. falciparum* VAR2CSA induces an anti-tumour effect in Burkitt lymphoma by targeting oncofetal chondroitin sulphate, which is highly expressed in Burkitt lymphoma (40).

Limited studies indicate that parasite-derived molecules can exert biological effects in cancer models beyond the parasite’s original infection niche, supporting their potential as novel therapeutics. For instance, *T. spiralis* ES L1 in melanoma (36) and *T. gondii*-derived peptides in colorectal and hepatocellular cancer models (50). This provides preliminary evidence that some parasite-derived products could be explored as potential therapeutic or mechanistic tools in cancer research.

Immunosuppression resulting from infectious diseases, malnutrition, or chemotherapy increases susceptibility to opportunistic parasitic infections (77). Compromised immune function may allow persistent parasitic infections, leading to chronic inflammation, tissue damage, and disrupted immune signalling, which can contribute to cancer development and disease progression. The prevalence of intestinal parasitic infections such as *Cryptosporidium* spp. and *T. trichiura* amongst HIV patients is 19.6%, highlighting the importance of regular screening and treatment for intestinal parasitic infections in individuals with HIV/AIDS (78). Moreover, a previous systematic review and meta-analysis found that 28.4% of cancer patients had intestinal parasites, mainly *B. hominis* and *C. parvum* (79). Another systematic review found that 19.7% of CRC patients had intestinal parasites such as *Cryptosporidium* spp. and a much higher risk (OR 3.61) of developing CRC than healthy people (80). Here, a study showed that a low dose of *C. parvum* oocysts, confirmed by qPCR, induces digestive adenocarcinoma (33). Persistent *C. parvum* infection promotes an immunosuppressive tumour environment through immunosuppressive cells and pro-inflammatory mediators (49).

Several limitations impact the interpretation of these findings. Most notably, the predominance of dated *in vivo* studies may reflect the technical challenges of parasite–tumour co-infection models, ethical restrictions, high costs, and host variability. The reviewed research showed significant methodological differences, including the use of various parasite species or products, animal models, cell lines, carcinogens, and endpoints, making direct comparisons difficult. Furthermore, publication bias and the lack of extensive human clinical validation limit the applicability of the results, particularly regarding the therapeutic use of parasite-derived molecules. Humanised mouse models may enhance the translational relevance of *in vivo* parasite-cancer studies. Future epidemiological research combining parasitological and cancer incidence data could help establish human correlations. Additionally, detailed analysis and recombinant production of parasite proteins may be crucial for developing anti-tumour secretomes.

In conclusion, *in vitro* and *in vivo* studies indicate that parasites and parasite-derived proteins can exert protumour and antitumour effects, determined by parasite species, infection chronicity, and host immune context. Chronic infection can promote tumour development by inducing M2 macrophage polarisation, expanding Treg and Th2 cells, and sustaining the signalling of anti-inflammatory cytokines such as IL-10 and TGF-β. In addition, chronic infection leads to desmoplastic stromal remodelling, which supports immune evasion by cancer cells. In contrast, acute infection or infections that are immunologically resolved can activate tumour-killing immune responses. These responses include immune-mediated cytotoxicity, polarisation towards Th1 cells, induction of tumour cell apoptosis, and inhibition of tumour cell migration. Collectively, these findings underscore that the parasite-cancer relationship is governed by the microenvironment. This provides a rationale for investigating parasite-derived molecules as potential therapeutic candidates, although mechanistic precision is required prior to clinical translation.

## Data Availability

The original contributions presented in the study are included in the article/supplementary material. Further inquiries can be directed to the corresponding author.

## References

[1] Filho AM Laversanne M Ferlay J Colombet M Pineros M Znaor A . The GLOBOCAN 2022 cancer estimates: Data sources, methods, and a snapshot of the cancer burden worldwide. Int J Cancer. (2025) 156:1336–46. doi: 10.1002/ijc.35278 39688499

[2] Hyndman IJ . Review: the contribution of both nature and nurture to carcinogenesis and progression in solid tumours. Cancer Microenviron. (2016) 9:63–9. doi: 10.1007/s12307-016-0183-4 27066794 PMC4842185

[3] Wu S Zhu W Thompson P Hannun YA . Evaluating intrinsic and non-intrinsic cancer risk factors. Nat Commun. (2018) 9:3490. doi: 10.1038/s41467-018-05467-z 30154431 PMC6113228

[4] De Martel C Georges D Bray F Ferlay J Clifford GM . Global burden of cancer attributable to infections in 2018: a worldwide incidence analysis. Lancet Glob Health. (2020) 8:e180–90. doi: 10.1016/s2214-109x(19)30488-7 31862245

[5] Parkin DM . The global health burden of infection-associated cancers in the year 2002. Int J Cancer. (2006) 118:3030–44. doi: 10.1002/ijc.21731 16404738

[6] Dalton-Griffin L Kellam P . Infectious causes of cancer and their detection. J Biol. (2009) 8:67. doi: 10.1186/jbiol168 19678917 PMC2736673

[7] Samaras V Rafailidis PI Mourtzoukou EG Peppas G Falagas ME . Chronic bacterial and parasitic infections and cancer: a review. J Infect Dev Ctries. (2010) 4:267–81. doi: 10.3855/jidc.819 20539059

[8] Hewitson JP Grainger JR Maizels RM . Helminth immunoregulation: the role of parasite secreted proteins in modulating host immunity. Mol Biochem Parasitol. (2009) 167:1–11. doi: 10.1016/j.molbiopara.2009.04.008 19406170 PMC2706953

[9] Wu Z Wang L Tang Y Sun X . Parasite-derived proteins for the treatment of allergies and autoimmune diseases. Front Microbiol. (2017) 8:2164. doi: 10.3389/fmicb.2017.02164 29163443 PMC5682104

[10] Alghanmi M Minshawi F Altorki TA Zawawi A Alsaady I Naser AY . Helminth-derived proteins as immune system regulators: a systematic review of their promise in alleviating colitis. BMC Immunol. (2024) 25:21. doi: 10.1186/s12865-024-00614-2 38637733 PMC11025257

[11] El Skhawy N Shehata A Eissa MM . Parasites' immunomodulators: a breakthrough in immunotherapeutics displaying antineoplastic activity against human colorectal and hepatocellular carcinoma cells. Infect Agent Cancer. (2025) 21:5. doi: 10.1186/s13027-025-00715-6 41387891 PMC12805742

[12] Mani R Martin CG Balu KE Wang Q Rychahou P Izumi T . A novel protozoa parasite-derived protein adjuvant is effective in immunization with cancer cells to activate the cancer-specific protective immunity and inhibit the cancer growth in a murine model of colorectal cancer. Cells. (2024) 13:111. doi: 10.3390/cells13020111 38247803 PMC10814441

[13] Fried B Reddy A Mayer D . Helminths in human carcinogenesis. Cancer Lett. (2011) 305:239–49. doi: 10.1016/j.canlet.2010.07.008 20667649

[14] Callejas BE Martinez-Saucedo D Terrazas LI . Parasites as negative regulators of cancer. Biosci Rep. (2018) 38:BSR20180935. doi: 10.1042/bsr20180935 30266743 PMC6200699

[15] Van Tong H Brindley PJ Meyer CG Velavan TP . Parasite infection, carcinogenesis and human Malignancy. EBioMedicine. (2017) 15:12–23. doi: 10.1016/j.ebiom.2016.11.034 27956028 PMC5233816

[16] Pawlowska M Jarek D Milanowski J Szewczyk-Golec K . Parasitic infections and carcinogenesis: molecular mechanisms, immune modulation, and emerging therapeutic strategies. Oncol Res. (2026) 34:8. doi: 10.32604/or.2025.071891 PMC12848758 41613796

[17] Ding H Wu S Jin Z Zheng B Hu Y He K . Anti-tumor effect of parasitic protozoans. Bioengineering (Basel). (2022) 9:395. doi: 10.3390/bioengineering9080395 36004920 PMC9405343

[18] Thamavit W Bhamarapravati N Sahaphong S Vajrastira S Angsubhakorn S . Effects of dimethylnitrosamine on induction of cholangiocarcinoma in Opisthorchis viverrini-infected Syrian golden hamsters. Cancer Res. (1978) 38:4634–9. 214229

[19] Thamavit W Kongkanuntn R Tiwawech D Moore MA . Level of Opisthorchis infestation and carcinogen dose-dependence of cholangiocarcinoma induction in Syrian golden hamsters. Virchows Arch B Cell Pathol incl Mol Pathol. (1987) 54:52–8. doi: 10.1007/bf02899196 2892303

[20] Thamavit W Moore MA Hiasa Y Ito N . Generation of high yields of Syrian hamster cholangiocellular carcinomas and hepatocellular nodules by combined nitrite and aminopyrine administration and Opisthorchis viverrini infection. Jpn J Cancer Res. (1988) 79:909–16. doi: 10.1111/j.1349-7006.1988.tb00054.x 2846484 PMC5917610

[21] Moore MA Thamavit W Hiasa Y Ito N . Early lesions induced by DHPN in Syrian golden hamsters: influence of concomitant Opisthorchis infestation, dehydroepiandrosterone or butylated hydroxyanisole administration. Carcinogenesis. (1988) 9:1185–9. doi: 10.1093/carcin/9.7.1185 2968188

[22] Miyahara K Yokoo N Sakurai H Igarashi I Sakata Y Yoshida Y . Antitumor activity of Toxoplasma lysate antigen against methylcholanthrene-induced tumor-bearing rats. J Vet Med Sci. (1992) 54:221–8. doi: 10.1292/jvms.54.221 1606251

[23] Thamavit W Pairojkul C Tiwawech D Shirai T Ito N . Strong promoting effect of Opisthorchis viverrini infection on dimethylnitrosamine-initiated hamster liver. Cancer Lett. (1994) 78:121–5. doi: 10.1016/0304-3835(94)90040-x 8180954

[24] Thamavit W Tiwawech D Moore MA Ito N Shirai T . Equivocal evidence of complete carcinogenicity after repeated infection of Syrian hamsters with Opisthorchis viverrini. Toxicol Pathol. (1996) 24:493–7. doi: 10.1177/019262339602400412 8864191

[25] Moore MA Thamavit W Tiwawech D Ito N Tsuda H . Modulation of dihydroxy-di-n-propylnitrosamine-induced liver lesion development in Opisthorchis-infected Syrian hamsters by praziquantel treatment in association with butylated hydroxyanisole or dehydroepiandrosterone administration. Jpn J Cancer Res. (1998) 89:1113–7. 10.1111/j.1349-7006.1998.tb00505.x PMC5921716 9914779

[26] Oliveira EC Leite MS Miranda JA Andrade AL Garcia SB Luquetti AO . Chronic Trypanosoma cruzi infection associated with low incidence of 1,2-dimethylhydrazine-induced colon cancer in rats. Carcinogenesis. (2001) 22:737–40. doi: 10.1093/carcin/22.5.737 11323392

[27] Boonmars T Wu Z Boonjaruspinyo S Pinlaor S Nagano I Takahashi Y . Alterations of gene expression of RB pathway in Opisthorchis viverrini infection-induced cholangiocarcinoma. Parasitol Res. (2009) 105:1273–81. doi: 10.1007/s00436-009-1548-0 19582476

[28] Botelho M Oliveira P Gomes J Gartner F Lopes C da Costa JM . Tumourigenic effect of Schistosoma haematobium total antigen in mammalian cells. Int J Exp Pathol. (2009) 90:448–53. doi: 10.1111/j.1365-2613.2009.00650.x PMC2741155 19659903

[29] Botelho M Ferreira AC Oliveira MJ Domingues A MaChado JC da Costa JM . Schistosoma haematobium total antigen induces increased proliferation, migration and invasion, and decreases apoptosis of normal epithelial cells. Int J Parasitol. (2009) 39:1083–91. doi: 10.1016/j.ijpara.2009.02.016 19285502

[30] Wu Z Boonmars T Boonjaraspinyo S Nagano I Pinlaor S Puapairoj A . Candidate genes involving in tumorigenesis of cholangiocarcinoma induced by Opisthorchis viverrini infection. Parasitol Res. (2011) 109:657–73. doi: 10.1007/s00436-011-2298-3 21380578

[31] Botelho MC Oliveira PA Lopes C Correia da Costa JM MaChado JC . Urothelial dysplasia and inflammation induced by Schistosoma haematobium total antigen instillation in mice normal urothelium. Urol Oncol. (2011) 29:809–14. doi: 10.1016/j.urolonc.2009.09.017 19945304

[32] Chan KH Chandramathi S Suresh K Chua KH Kuppusamy UR . Effects of symptomatic and asymptomatic isolates of Blastocystis hominis on colorectal cancer cell line, HCT116. Parasitol Res. (2012) 110:2475–80. doi: 10.1007/s00436-011-2788-3 22278727

[33] Benamrouz S Guyot K Gazzola S Mouray A Chassat T Delaire B . Cryptosporidium parvum infection in SCID mice infected with only one oocyst: qPCR assessment of parasite replication in tissues and development of digestive cancer. PloS One. (2012) 7:e51232. doi: 10.1371/journal.pone.0051232 23272093 PMC3521773

[34] Botelho MC Vale N Gouveia MJ Rinaldi G Santos J Santos LL . Tumour-like phenotypes in urothelial cells after exposure to antigens from eggs of Schistosoma haematobium: an oestrogen-DNA adducts mediated pathway? Int J Parasitol. (2013) 43:17–26. doi: 10.1016/j.ijpara.2012.10.023 23260770

[35] El-Tonsy MM Hussein HM Helal Tel S Tawfik RA Koriem KM Hussein HM . Schistosoma mansoni infection: is it a risk factor for development of hepatocellular carcinoma? Acta Trop. (2013) 128:542–7. doi: 10.1016/j.actatropica.2013.07.024 23932944

[36] Vasilev S Ilic N Gruden-Movsesijan A Vasilijic S Bosic M Sofronic-Milosavljevic L . Necrosis and apoptosis in Trichinella spiralis-mediated tumour reduction. Cent Eur J Immunol. (2015) 40:42–53. doi: 10.5114/ceji.2015.50832 26155183 PMC4472539

[37] Smout MJ Sotillo J Laha T Papatpremsiri A Rinaldi G Pimenta RN . Carcinogenic parasite secretes growth factor that accelerates wound healing and potentially promotes neoplasia. PloS Pathog. (2015) 11:e1005209. doi: 10.1371/journal.ppat.1005209 26485648 PMC4618121

[38] Chaiyadet S Sotillo J Smout M Cantacessi C Jones MK Johnson MS . Carcinogenic liver fluke secretes extracellular vesicles that promote cholangiocytes to adopt a tumorigenic phenotype. J Infect Dis. (2015) 212:1636–45. doi: 10.1093/infdis/jiv291 25985904 PMC4621255

[39] Liao CC Song EJ Chang TY Lin WC Liu HS Chen LR . Evaluation of cellular retinoic acid binding protein 2 gene expression through the retinoic acid pathway by co-incubation of Blastocystis ST-1 with HT29 cells *in vitro*. Parasitol Res. (2016) 115:1965–75. doi: 10.1007/s00436-016-4939-z 26911149

[40] Agerbaek MO Pereira MA Clausen TM Pehrson C Oo HZ Spliid C . Burkitt lymphoma expresses oncofetal chondroitin sulfate without being a reservoir for placental malaria sequestration. Int J Cancer. (2017) 140:1597–608. doi: 10.1002/ijc.30575 PMC5318225 27997697

[41] Chaiyadet S Smout M Laha T Sripa B Loukas A Sotillo J . Proteomic characterization of the internalization of Opisthorchis viverrini excretory/secretory products in human cells. Parasitol Int. (2017) 66:494–502. doi: 10.1016/j.parint.2016.02.001 26873540 PMC5149449

[42] Haugen B Karinshak SE Mann VH Popratiloff A Loukas A Brindley PJ . Granulin secreted by the food-borne liver fluke Opisthorchis viverrini promotes angiogenesis in human endothelial cells. Front Med (Lausanne). (2018) 5:30. doi: 10.3389/fmed.2018.00030 29503819 PMC5820972

[43] Staff PNTD . Correction: Connexin 43 plays an important role in the transformation of cholangiocytes with Clonochis sinensis excretory-secretory protein and N-nitrosodimethylamine. PloS NeglTrop Dis. (2019) 13:e0007526. doi: 10.1371/journal.pntd.0007526 31251742 PMC6599143

[44] Eissa MM Ismail CA El-Azzouni MZ Ghazy AA Hadi MA . Immuno-therapeutic potential of Schistosoma mansoni and Trichinella spiralis antigens in a murine model of colon cancer. Invest New Drugs. (2019) 37:47–56. doi: 10.1007/s10637-018-0609-6 29808307

[45] Lin Y Zhu S Hu C Wang J Jiang P Zhu L . Cross-species suppression of hepatoma cell growth and migration by a Schistosoma japonicum microRNA. Mol Ther Nucleic Acids. (2019) 18:400–12. doi: 10.1016/j.omtn.2019.09.006 31655260 PMC6831938

[46] Ruiz-Manzano RA Palacios-Arreola MI Hernandez-Cervantes R Del Rio-Araiza VH Nava-Castro KE Ostoa-Saloma P . Potential novel risk factor for breast cancer: Toxocara canis infection increases tumor size due to modulation of the tumor immune microenvironment. Front Oncol. (2020) 10:736. doi: 10.3389/fonc.2020.00736 32547942 PMC7272683

[47] Weglage J Wolters F Hehr L Lichtenberger J Wulz C Hempel F . Schistosoma mansoni eggs induce Wnt/beta-catenin signaling and activate the protooncogene c-Jun in human and hamster colon. Sci Rep. (2020) 10:22373. doi: 10.1038/s41598-020-79450-4 33361772 PMC7758332

[48] Hu C Li Y Pan D Wang J Zhu L Lin Y . A Schistosoma japonicum microRNA exerts antitumor effects through inhibition of both cell migration and angiogenesis by targeting PGAM1. Front Oncol. (2021) 11:652395. doi: 10.3389/fonc.2021.652395 34221971 PMC8242254

[49] Sawant M Benamrouz-Vanneste S Mouray A Bouquet P Gantois N Creusy C . Persistent Cryptosporidium parvum infection leads to the development of the tumor microenvironment in an experimental mouse model: Results of a microarray approach. Microorganisms. (2021) 9:2569. doi: 10.3390/microorganisms9122569 34946170 PMC8704780

[50] Shahrivar F Sadraei J Pirestani M Ahmadpour E . Enhancement of apoptosis in Caco-2, Hep-G2, and HT29 cancer cell lines following exposure to Toxoplasma gondii peptides. Drug Target Insights. (2024) 18:70–7. doi: 10.33393/dti.2024.3177 39355763 PMC11443429

[51] Bellini I Scribano D Ambrosi C Chiovoloni C Rondon S Pronio A . Anisakis extracellular vesicles elicit immunomodulatory and potentially tumorigenic outcomes on human intestinal organoids. Parasit Vectors. (2024) 17:393. doi: 10.1186/s13071-024-06471-7 39285481 PMC11406850

[52] Reddy KB Nabha SM Atanaskova N . Role of MAP kinase in tumor progression and invasion. Cancer Metastasis Rev. (2003) 22:395–403. doi: 10.1023/a:1023781114568 12884914

[53] Park MH Hong JT . Roles of NF-kappaB in cancer and inflammatory diseases and their therapeutic approaches. Cells. (2016) 5. doi: 10.3390/cells5020015 27043634 PMC4931664

[54] Qu K Wang Z Fan H Li J Liu J Li P . Correction to: MCM7 promotes cancer progression through cyclin D1-dependent signaling and serves as a prognostic marker for patients with hepatocellular carcinoma. Cell Death Dis. (2022) 13:950. doi: 10.1038/s41419-022-05405-4 36357373 PMC9649803

[55] Sripa B Deenonpoe R Brindley PJ . Co-infections with liver fluke and Helicobacter species: a paradigm change in pathogenesis of opisthorchiasis and cholangiocarcinoma? Parasitol Int. (2017) 66:383–9. doi: 10.1016/j.parint.2016.11.016 27919744 PMC5457716

[56] Thorley-Lawson D Deitsch KW Duca KA Torgbor C . The link between Plasmodium falciparum malaria and endemic Burkitt's lymphoma-new insight into a 50-year-old enigma. PloS Pathog. (2016) 12:e1005331. doi: 10.1371/journal.ppat.1005331 26794909 PMC4721646

[57] Sana B Nedjoud G Sihem K Hadia H Aslam AA Mughram MHA . N-nitrosodimethylamine as an emerging environmental contaminant: Sources, analytical advances, and ecotoxicological and human health risks. Int J Anal Chem. (2026) 2026:1536227. doi: 10.1155/ianc/1536227 41953580 PMC13055445

[58] Migasena P Reaunsuwan W Changbumrung S . Nitrates and nitrites in local Thai preserved protein foods. J Med Assoc Thai. (1980) 63:500–5. 7420001

[59] Thamavit W Moore MA Hiasa Y Ito N . Enhancement of DHPN induced hepatocellular, cholangiocellular and pancreatic carcinogenesis by Opisthorchis viverrini infestation in Syrian golden hamsters. Carcinogenesis. (1988) 9:1095–8. doi: 10.1093/carcin/9.6.1095 2836105

[60] Wagner M Nishikawa H Koyasu S . Reinventing type 2 immunity in cancer. Nature. (2025) 637:296–303. doi: 10.1038/s41586-024-08194-2 39780006

[61] Wagner M Koyasu S . Cancer in disguise: a parasite within. EMBO J. (2026) 45:1051–9. doi: 10.1038/s44318-025-00691-y 41530388 PMC12909802

[62] Zaiss DMW Pearce EJ Artis D McKenzie ANJ Klose CSN . Cooperation of ILC2s and T(H)2 cells in the expulsion of intestinal helminth parasites. Nat Rev Immunol. (2024) 24:294–302. doi: 10.1038/s41577-023-00942-1 37798539

[63] Xu J Ding L Mei J Hu Y Kong X Dai S . Dual roles and therapeutic targeting of tumor-associated macrophages in tumor microenvironments. Signal Transduct Target Ther. (2025) 10:268. doi: 10.1038/s41392-025-02325-5 40850976 PMC12375796

[64] Wynn TA Vannella KM . Macrophages in tissue repair, regeneration, and fibrosis. Immunity. (2016) 44:450–62. doi: 10.1016/j.immuni.2016.02.015 26982353 PMC4794754

[65] Gause WC Wynn TA Allen JE . Type 2 immunity and wound healing: evolutionary refinement of adaptive immunity by helminths. Nat Rev Immunol. (2013) 13:607–14. doi: 10.1038/nri3476 23827958 PMC3789590

[66] Dvorak HF . Tumors: wounds that do not heal-redux. Cancer Immunol Res. (2015) 3:1–11. doi: 10.1158/2326-6066.cir-14-0209 25568067 PMC4288010

[67] Legrand F Driss V Delbeke M Loiseau S Hermann E Dombrowicz D . Human eosinophils exert TNF-alpha and granzyme A-mediated tumoricidal activity toward colon carcinoma cells. J Immunol. (2010) 185:7443–51. doi: 10.4049/jimmunol.1000446 21068403

[68] Nielsen HJ Hansen U Christensen IJ Reimert CM Brunner N Moesgaard F . Independent prognostic value of eosinophil and mast cell infiltration in colorectal cancer tissue. J Pathol. (1999) 189:487–95. doi: 10.1002/(sici)1096-9896(199912)189:4<487::aid-path484>3.0.co;2-i 10629548

[69] Hu G Wang S Zhong K Xu F Huang L Chen W . Tumor-associated tissue eosinophilia predicts favorable clinical outcome in solid tumors: a meta-analysis. BMC Cancer. (2020) 20:454. doi: 10.1186/s12885-020-06966-3 32434481 PMC7240929

[70] Simson L Ellyard JI Dent LA Matthaei KI Rothenberg ME Foster PS . Regulation of carcinogenesis by IL-5 and CCL11: a potential role for eosinophils in tumor immune surveillance. J Immunol. (2007) 178:4222–9. doi: 10.4049/jimmunol.178.7.4222 17371978

[71] Tepper RI Pattengale PK Leder P . Murine interleukin-4 displays potent anti-tumor activity *in vivo*. Cell. (1989) 57:503–12. doi: 10.1016/0092-8674(89)90925-2 2785856

[72] Feng B Bai Z Zhou X Zhao Y Xie YQ Huang X . The type 2 cytokine Fc-IL-4 revitalizes exhausted CD8(+) T cells against cancer. Nature. (2024) 634:712–20. doi: 10.1038/s41586-024-07962-4 39322665 PMC11485240

[73] Asghari A Nourmohammadi H Majidiani H Shariatzadeh SA Anvari D Shamsinia S . Promising effects of parasite-derived compounds on tumor regression: a systematic review of *in vitro* and *in vivo* studies. Environ Sci pollut Res Int. (2022) 29:32383–96. doi: 10.1007/s11356-021-17090-5 35146610

[74] Aslam A Refaat B Almaimani RA Obaid AA Mujalli A Farrash WF . Increased protein expression of interleukin-10 and its signalling molecules in colon cancer progression: potential prognostic and therapeutic targets. Discov Oncol. (2025) 16:637. doi: 10.1007/s12672-025-02452-z 40299210 PMC12040807

[75] Zhu J Xu Z Chen X Zhou S Zhang W Chi Y . Parasitic antigens alter macrophage polarization during Schistosoma japonicum infection in mice. Parasit Vectors. (2014) 7:122. doi: 10.1186/1756-3305-7-122 24666892 PMC3975460

[76] Whittle HC Brown J Marsh K Greenwood BM Seidelin P Tighe H . T-cell control of Epstein-Barr virus-infected B cells is lost during P. falciparum malaria. Nature. (1984) 312:449–50. doi: 10.1038/312449a0 6095104

[77] Evering T Weiss LM . The immunology of parasite infections in immunocompromised hosts. Parasite Immunol. (2006) 28:549–65. doi: 10.1111/j.1365-3024.2006.00886.x 17042927 PMC3109637

[78] Seema K Kumar A Boipai M Kumar M Sharma AK . Prevalence of intestinal parasites in HIV/AIDS-infected patients with correlation to CD4+ T-cell count at hospital in Eastern India. J Family Med Prim Care. (2023) 12:2884–7. doi: 10.4103/jfmpc.jfmpc_806_23 38186830 PMC10771173

[79] Wondmagegn YM Setegn A Girmay G Abebe W Dessie N Ashagre A . Global prevalence of intestinal parasites in cancer patients: a systematic review and meta-analysis. BMC Infect Dis. (2025) 25:815. doi: 10.1186/s12879-025-11207-8 40596954 PMC12211656

[80] Hataminejad M Basirpour B Baharlou M Gholami Koohestan M Ziaei Hezarjaribi H Rahimi Esboei B . Global prevalence and correlation of intestinal parasitic infections in patients with colorectal cancer: a systematic review and meta-analysis. BMC Gastroenterol. (2025) 25:584. doi: 10.1186/s12876-025-04144-y 40804378 PMC12351808

